# Comparative Evaluation of the Shear Bond Strength of Metallic and Ceramic Brackets Bonded Using Seventh- and Eighth-Generation Bonding Agents: An In Vitro Study

**DOI:** 10.7759/cureus.91698

**Published:** 2025-09-06

**Authors:** Hemannthee Thota, Deekshitha Achanta, Kiran Kumar Dodda, Rajapatruni Supriya, Chaitanya Krishna Dev Pillutla, Revathi Peddu

**Affiliations:** 1 Department of Orthodontics, Sibar Institute of Dental Sciences, Guntur, IND; 2 Department of Orthodontics, Dr. Gowds Dental Hospital, Hyderabad, IND; 3 Department of Orthodontics, Drs. Sudha and Nageswara Rao Siddhartha Institute of Dental Sciences, Vijayawada, IND; 4 Department of Orthodontics, GITAM Dental College & Hospital, Vishakhapatnam, IND

**Keywords:** bonding agents, ceramic brackets, eighth-generation adhesive, instron universal testing machine, in vitro study, metal brackets, self-etch, seventh-generation adhesive, shear bond strength

## Abstract

Objective

This in vitro study aims to compare the shear bond strength (SBS) of metal and ceramic orthodontic brackets bonded using seventh- and eighth-generation bonding agents.

Materials and methods

Eighty extracted premolars were cleaned with pumice, rinsed with distilled water, air-dried, and embedded in acrylic resin. The samples were divided into four groups (n = 20). Group A consisted of 3M Unitek™ metal brackets (3M Company, Monrovia, CA, USA) bonded with 3M ESPE Single Bond Universal (seventh generation). Group B included 3M Unitek ceramic brackets bonded with 3M ESPE Single Bond Universal. Group C comprised 3M Unitek metal brackets bonded with GC G-Premio BOND (eighth generation, GC Corporation, Tokyo, Japan), and Group D included 3M Unitek ceramic brackets bonded with GC G-Premio Bond. Brackets were bonded with 3M ESPE composite under dry conditions at room temperature. The bonded samples were stored in artificial saliva at 37 °C for 24 hours, and SBS was measured using an Instron universal testing machine.

Results

The SBS for metal brackets bonded with 3M ESPE Single Bond Universal was 11.54 ± 0.91 MPa, while ceramic brackets with the same adhesive showed 13.42 ± 0.80 MPa. For metal brackets bonded with GC G-Premio Bond, the SBS was 13.09 ± 0.79 MPa, and for ceramic brackets, it was 15.17 ± 0.98 MPa. One-way ANOVA revealed statistically significant differences among the groups (p = 0.0001).

Conclusions

The findings indicate that both adhesive type and bracket material significantly influence bond strength. Eighth-generation adhesives outperformed seventh-generation adhesives, and ceramic brackets demonstrated higher bond strength than metal brackets. All SBS values exceeded Reynolds’ clinical threshold, confirming the adequacy of both adhesives. The superior performance and simplified application of the eighth-generation adhesive support its effectiveness in contemporary orthodontic practice.

## Introduction

The introduction of acid etching by Buonocore in 1955 marked a transformative moment in orthodontics, laying the foundation for modern adhesive techniques [1]. This was followed by Bowen’s development of Bis-GMA resin in 1956, which became integral to dental composites by 1962 [2]. Newman’s introduction of direct bonding in 1965 further revolutionized bracket adhesion, incorporating these advances into clinical practice [3].

Over the decades, orthodontic adhesives have evolved through multiple generations, with the goals of enhancing treatment efficiency, improving bond strength, and preserving enamel integrity. A key parameter for evaluating these materials is shear bond strength (SBS), with Reynolds recommending values between 5.9 and 7.8 MPa to ensure adequate bracket retention without risking enamel damage during debonding [4].

Among modern systems, seventh-generation adhesives (one-step self-etch) simplify clinical procedures by combining etching, priming, and bonding into a single step. However, their relatively mild self-etching capability may result in insufficient enamel etching, potentially leading to lower SBS in orthodontic applications. In contrast, eighth-generation or universal adhesives allow for multiple application modes, such as self-etch, total-etch, or selective-etch, providing clinicians with greater flexibility. These systems often incorporate functional monomers such as 10-methacryloyloxydecyl dihydrogen phosphate, which can form stable chemical bonds with hydroxyapatite in enamel and dentin, thereby enhancing both immediate and long-term bond strength. They also demonstrate improved moisture tolerance, a critical advantage during intraoral procedures [5]. Despite these advantages, evidence directly comparing the orthodontic performance of seventh- and eighth-generation adhesives, particularly with different bracket types, remains limited. Therefore, the aim of this study is to evaluate and compare the SBS of orthodontic brackets bonded to enamel using seventh- and eighth-generation adhesive systems across both metal and ceramic brackets.

Although in vitro testing cannot fully replicate the oral environment, it provides valuable and controlled conditions for assessing bonding performance. This comparison is clinically relevant, as it supports orthodontists in selecting bonding protocols that improve bracket retention, reduce bond failures, and optimize treatment outcomes. Building on the foundational work of Bark Meir and Cooley, this study contributes to the ongoing development of safer and more effective orthodontic bonding techniques [6].

## Materials and methods

Study design and sample size determination

An a priori power analysis was conducted using G*Power (version 3.1.9.4) for a fixed-effects, one-way ANOVA (F tests → ANOVA: fixed effects, omnibus, one-way), with an alpha level of 0.05, power of 0.80, four groups, and a large effect size (f = 0.40). The effect size was derived from Sharma et al. (2014), who compared SBS (the dependent variable, measured in megapascals (MPa)) among four different orthodontic adhesives [7]. The analysis indicated a minimum total sample size of 76 specimens (n = 19 per group). To enhance statistical power and account for potential specimen loss, the sample size was rounded up to 80 (n = 20 per group).

This in vitro study used 80 extracted maxillary and mandibular premolars collected from patients undergoing extractions for orthodontic, periodontal, or prosthetic reasons. The study was conducted in the Department of Orthodontics and Dentofacial Orthopedics, Drs. Sudha and Nageswara Rao Siddhartha Institute of Dental Sciences, Vijayawada, India, over a two-month period (January to March 2024). Ethical approval was obtained from the Institutional Ethical Committee (approval number IEC/DRS.S&NRSIDS/2022/PG/18).

Inclusion and exclusion criteria

Inclusion criteria were extracted maxillary and mandibular premolars with intact enamel, free of cracks, caries, or restorations, while exclusion criteria included teeth with caries, cracks, fractures, restorations, or developmental disturbances.

Tooth preparation and bonding procedure

The extracted teeth were stored in distilled water at room temperature, with the water changed regularly to prevent bacterial growth and enamel degradation. Prior to bonding, the buccal surfaces were polished with pumice slurry (Garreco® Dental Pumice, Garreco LLC, Heber Springs, AR, USA), rinsed, and dried. Enamel etching was performed using 37% phosphoric acid (Scotchbond™ Universal Adhesive, 3M Company, St. Paul, MN, USA) for 15 seconds, followed by inspection for a frosty white appearance to confirm adequate etching.

**Figure 1:**
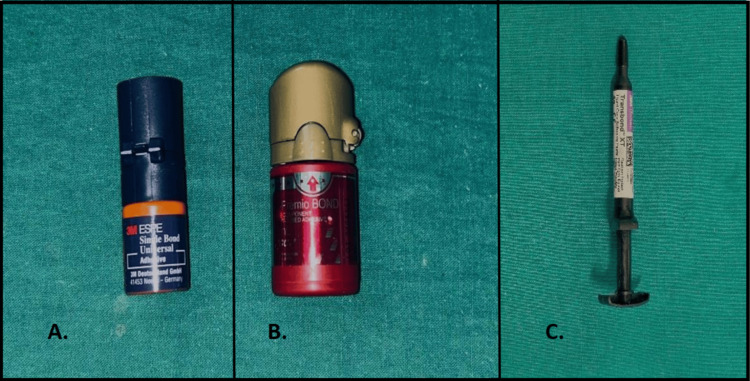
(A) 3M ESPE Single Bond Universal. (B) GC G-Premio Bond. (C) 3M ESPE Composite.

The bonding agents used in this study included a seventh-generation adhesive (Single Bond Universal, 3M ESPE, 3M Company) and an eighth-generation adhesive (G-Premio BOND, GC Corporation, Tokyo, Japan). Transbond™ XT (3M ESPE, 3M Company) was used to bond the brackets to the enamel surface (Figure 1). The metal and ceramic brackets were both obtained from 3M Unitek™ (3M Company, Monrovia, CA, USA). All brackets had identical torque and angulation values to ensure consistency and eliminate variations related to base design.

Group Allocation and Bonding Procedure

The teeth were randomly assigned to four groups using a simple randomization method to ensure unbiased distribution based on bracket type and bonding agent: Group A, 3M Unitek metal brackets with a seventh-generation adhesive; Group B, 3M Unitek ceramic brackets with a seventh-generation adhesive; Group C, 3M Unitek metal brackets with an eighth-generation adhesive; and Group D, 3M Unitek ceramic brackets with an eighth-generation adhesive.

**Figure 2:**
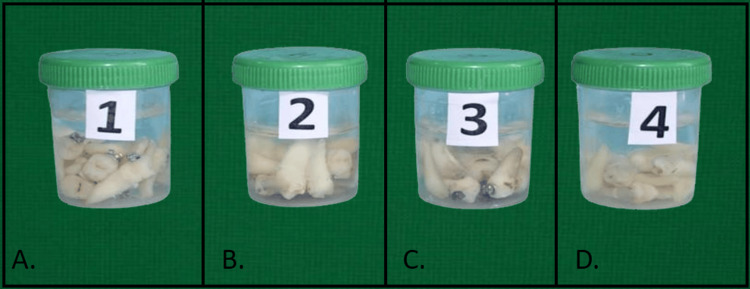
Extracted teeth divided into four groups, bonded, and placed in artificial saliva. (A) Metal brackets with seventh-generation adhesive. (B) Ceramic brackets with seventh-generation adhesive. (C) Metal brackets with eighth-generation adhesive. (D) Ceramic brackets with eighth-generation adhesive.

Brackets were bonded using 3M ESPE composite in a dry field and stored in artificial saliva for 24 hours post-bonding (Figure 2). In this study, laboratory-prepared artificial saliva was used to simulate oral conditions. The solution was formulated using standard components, such as sodium chloride, potassium chloride, calcium chloride dihydrate, sodium dihydrogen phosphate, disodium hydrogen phosphate, mucin, and urea, dissolved in distilled water to closely replicate the electrolyte composition of natural saliva (Table 1).

**Table 1:** Lab-synthesized artificial saliva composition for in vitro studies (per 1000 mL).

Component	Chemical formula	Concentration (g/L)	Purpose
Sodium chloride	NaCl	0.4	Maintains ionic strength
Potassium chloride	KCl	0.4	Provides potassium ions
Calcium chloride dihydrate	CaCl₂·2H₂O	0.795	Source of calcium for remineralization
Sodium dihydrogen phosphate	NaH₂PO₄·2H₂O	0.69	Buffering agent
Disodium hydrogen phosphate	Na₂HPO₄·2H₂O	0.866	Buffering agent
Urea	CO(NH₂)₂	1	Simulates metabolic nitrogen
Mucin (porcine/bovine)	-	2.000-5.000	Provides viscosity
Distilled water	H₂O	Up to 1000 mL	Solvent

Mounting and shear bond strength testing

**Figure 3:**
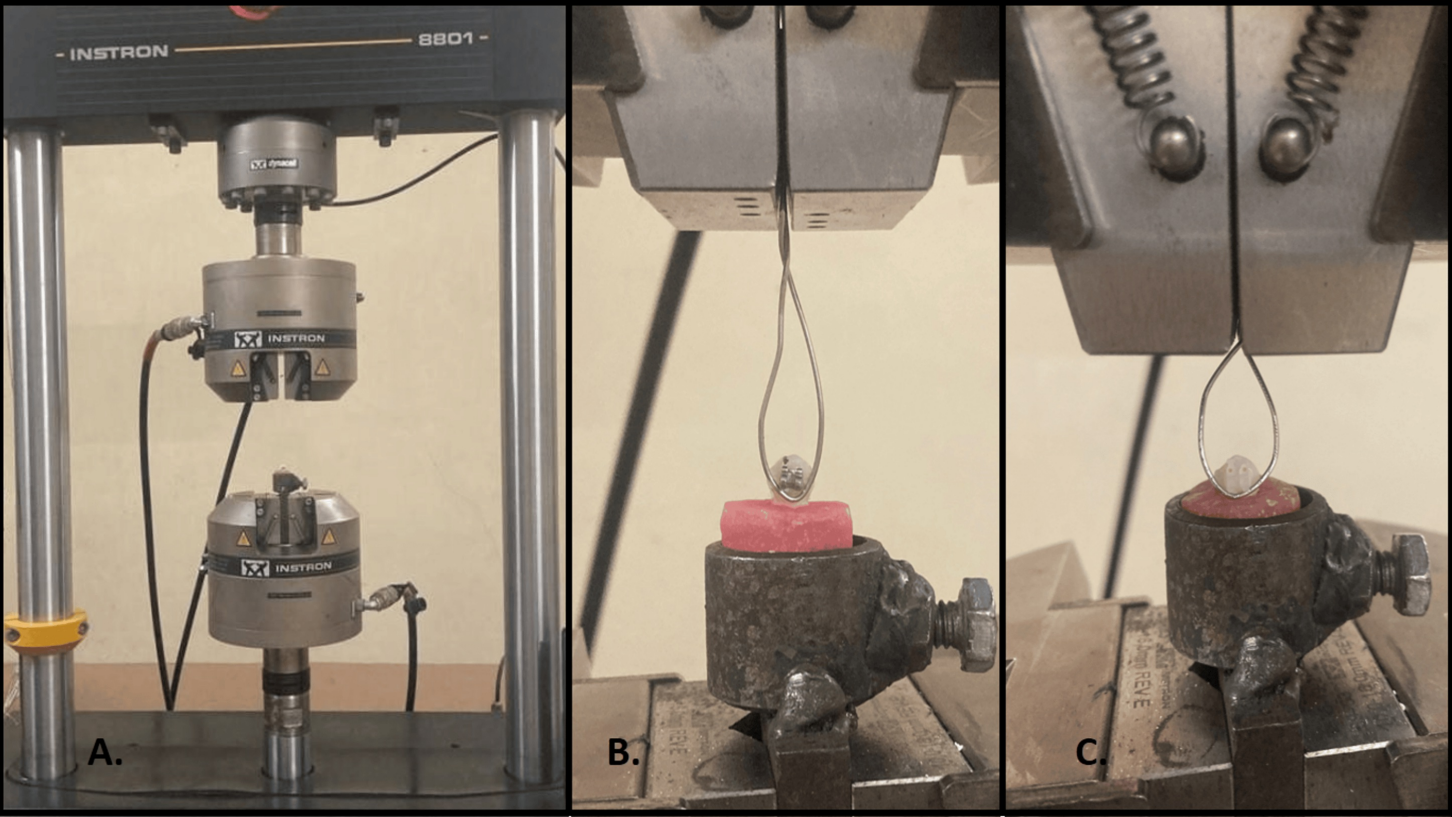
(A) Instron Universal Testing Machine. (B) SBS testing on metal brackets. (C) SBS testing on ceramic brackets. SBS, shear bond strength

Each tooth was mounted in self-cure acrylic blocks (Orthoresin, Dentsply Sirona, York, PA, USA) measuring 18 mm in diameter and 25 mm in height, embedding the root up to the cemento-enamel junction with the buccal surface aligned parallel to the block face. SBS testing was carried out using an Instron Universal Testing Machine (Model F 100 K, Instron Corporation, Norwood, MA, USA). The machine was calibrated according to the manufacturer’s guidelines prior to testing, and calibration was verified using certified standard weights to ensure force measurement accuracy within ±0.5%. The crosshead speed was maintained at 1 mm/min in accordance with ISO standards for bond strength testing. Routine calibration checks were performed throughout the study to ensure the reliability and validity of the force measurements (Figure 3).

Shear force was applied from the cervical to the occlusal direction, parallel to the bracket surface. The force at debonding was recorded in newtons (N) and converted to megapascals (MPa) using the following formula:

\[\text{Bond strength (MPa)} = \frac{\text{De-bonding force (N)}}{\text{Bracket surface area (mm}^2\text{)}}\]

**Figure 4:**
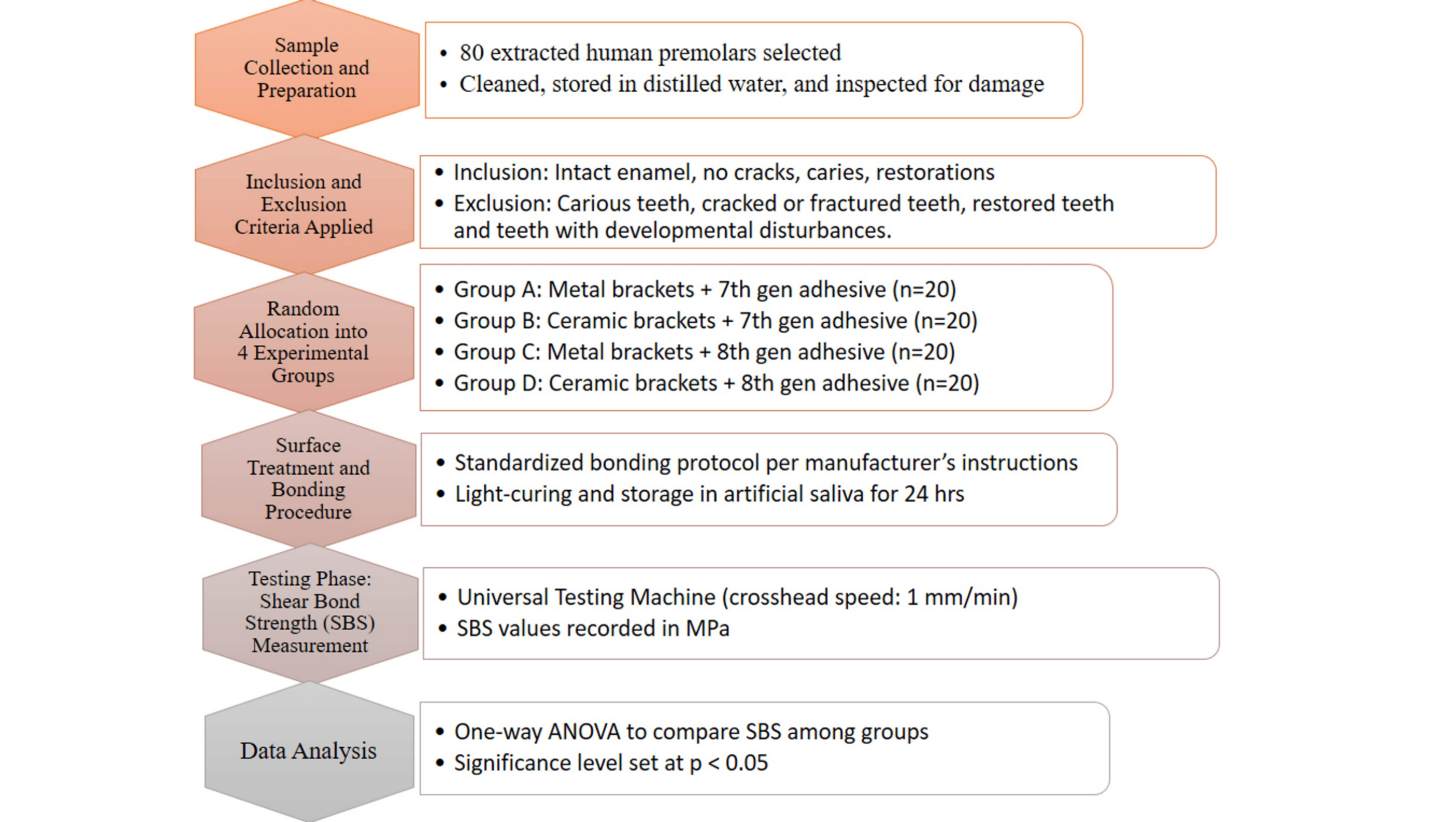
Study flowchart aligned with STROBE and EQUATOR EQUATOR, Enhancing the QUAlity and Transparency Of health Research; STROBE, STrengthening the Reporting of OBservational studies in Epidemiology

All samples were labeled appropriately and tested under uniform conditions to ensure consistency. An overview of the study methodology is presented in Figure 4.

Statistical analysis

Statistical analysis was performed using IBM SPSS Statistics for Windows, Version 26.0 (Released 2018; IBM Corp., Armonk, NY, USA). G*Power was used for sample size determination. Frequency, mean, and mode were used to provide a descriptive analysis of the data. The normality of SBS within each group (n = 20) was assessed using the Shapiro-Wilk test, and homogeneity of variances was evaluated with Levene’s test. As both assumptions were met (p > 0.05), a fixed-effects one-way ANOVA was conducted to compare the four groups. Post hoc pairwise comparisons were performed using Tukey’s honestly significant difference (HSD) test, with adjustments for multiple testing. Effect sizes for pairwise comparisons were reported as Cohen’s d. Statistical significance was set at α = 0.05.

## Results

A total of 80 samples were equally distributed across four groups (n = 20 per group). The mean SBS values for each group are presented in Table 2. Group A (metal brackets with a seventh-generation adhesive) recorded the lowest SBS (11.54 ± 0.91 MPa), whereas Group D (ceramic brackets with an eighth-generation adhesive) recorded the highest SBS (15.17 ± 0.98 MPa). 

**Table 2:** Mean SBS (MPa) ± SD in each group (n = 20; 25% of total sample)

Group	Bracket type + adhesive	Mean (MPa)	SD
A	Metal + seventh generation	11.54	0.91
B	Ceramic + seventh generation	13.42	0.80
C	Metal + eighth generation	13.09	0.79
D	Ceramic + eighth generation	15.17	0.98

All groups satisfied the assumptions of normality (Shapiro-Wilk test, p > 0.05 for each group) and homogeneity of variances (Levene’s test: F = 1.18, p = 0.325). The results are summarized in Table 3. Therefore, a one-way ANOVA was performed.

**Table 3:** Normality test (Shapiro-Wilk) All groups were normally distributed (p > 0.05).

Group	W statistic	p-Value
Group A	0.959	0.505
Group B	0.973	0.852
Group C	0.968	0.744
Group D	0.965	0.676

A one-way ANOVA revealed a statistically significant difference among the four groups, F(3, 76) = 57.91, p < 0.001. Post hoc pairwise comparisons using Tukey’s HSD test were performed to examine specific group differences. The results are summarized in Table 4.

**Table 4:** Tukey HSD post hoc comparison of SBS (MPa) Each group contributed 25% of the total sample (N = 80), with 20 specimens per group. Results indicate p < 0.001, which is considered highly significant. The F-value obtained from the one-way ANOVA was 57.912. HSD, honestly significant difference; SBS, shear bond strength

Intergroup comparison	Mean difference	Adjusted p-value	95% CI for mean	Significant
A vs. B	1.89	<0.001	1.16 to 2.61	Yes
A vs. C	1.56	<0.001	0.83 to 2.28	Yes
A vs. D	3.64	<0.001	2.91 to 4.36	Yes
B vs. C	-0.33	0.631	-1.06 to 0.40	No
B vs. D	1.75	<0.001	1.02 to 2.48	Yes
C vs. D	2.08	<0.001	1.35 to 2.80	Yes

Ceramic brackets bonded with the eighth-generation adhesive (Group D) demonstrated the highest SBS, which was significantly greater than that of all other groups. Metal brackets bonded with the seventh-generation adhesive (Group A) exhibited the lowest SBS. No significant difference was found between ceramic brackets with the seventh-generation adhesive (Group B) and metal brackets with the eighth-generation adhesive (Group C) (p = 0.631). All other intergroup comparisons were statistically significant (p < 0.001).

**Figure 5:**
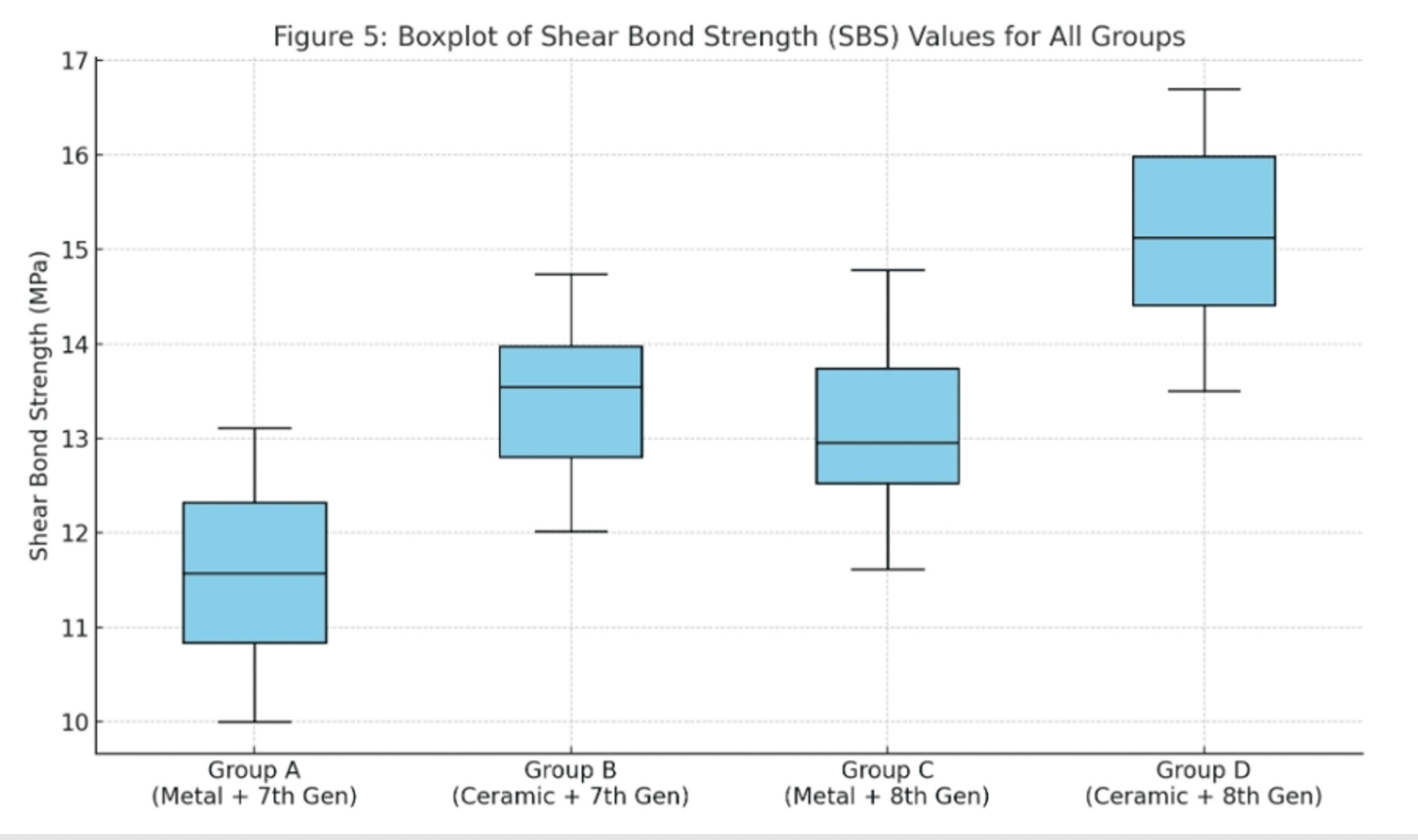
Box plot comparing SBS of metal and ceramic brackets bonded with seventh- and eighth-generation adhesives The plot illustrates the distribution of SBS values for each group, showing the IQR, median, and whiskers. SBS, shear bond strength

The plot visually represents the median SBS values, IQRs, and the overall data spread through whiskers and potential outliers for each group. Group D (ceramic brackets with eighth-generation adhesive) showed the highest median and overall SBS values, with a relatively narrow IQR, indicating both greater strength and consistency. Group A (metal brackets with seventh-generation adhesive) exhibited the lowest median SBS and a slightly broader IQR, suggesting greater variability in performance. Groups B and C (ceramic brackets with seventh-generation adhesive and metal brackets with eighth-generation adhesive, respectively) fell between Groups A and D, demonstrating moderate SBS values with relatively tight distributions. No extreme outliers were observed, and the whiskers extended within expected ranges, supporting the reliability of the dataset. Overall, the box plot confirmed the significant differences in SBS observed in the statistical analysis, highlighting the influence of both bracket material and adhesive generation on bond strength distribution (Figure 5).

## Discussion

The effectiveness of orthodontic bonding to different surfaces relies heavily on the secure attachment of brackets or bands to tooth surfaces, where bond strength plays a critical role. In orthodontics, optimal bond strength is essential for the effectiveness and success of treatment. A strong bond between orthodontic brackets is necessary to ensure the efficient transfer of forces required for tooth movement and alignment. Along with the development of orthodontic adhesives, modern metal and ceramic brackets have become widely used. Metal brackets typically have a relatively small base area of 8.97 mm² with a foil mesh retentive surface. In contrast, patients with high esthetic demands often prefer ceramic brackets because of their transparency and reduced visibility, despite their larger bracket base area of 11.69 mm² [8].

Reynolds and Fraunhofer reported that the minimum required bond strength for bracket bonding to enamel is 5.9-7.8 MPa [4]. Endo et al. stated that clinically acceptable SBS values range from 6 to 8 MPa [9], while Barceló Santana et al. suggested an ideal range of 6-10 MPa [10]. Fuhrmann et al. demonstrated that clinical bond strength should be between 5 and 8 MPa [11].

Several factors influence bond strength, including the type and generation of adhesive system, the condition of the enamel surface, maintenance of a dry working field during bonding, and the clinician’s technique in bracket placement and adhesive curing. The goal of adhesive formulation is to balance convenience with strong bonding capability while ensuring safe adhesive removal without damaging enamel. Recently developed bonding agents are dual-cured, self-etching, nano-reinforced systems that produce comparable bond strengths to both dentin and enamel. With their mild pH, these agents are believed to reduce postoperative sensitivity. Mild self-etching adhesives also leave hydroxyapatite crystals available for chemical bonding with functional monomers to calcium, potentially enhancing interface stability [12].

Since metal and ceramic brackets are the most commonly used materials in routine orthodontic practice, the present study assessed the SBS of these brackets when bonded with newer-generation adhesives, specifically seventh- and eighth-generation bonding agents. Previous studies have shown that both generations provide clinically viable SBS.

In this study, the highest SBS was observed in ceramic brackets bonded with eighth-generation adhesive (Group D: 15.17 MPa), followed by ceramic brackets bonded with seventh-generation adhesive (Group B: 13.4 MPa), metal brackets with eighth-generation adhesive (Group C: 13.09 MPa), and metal brackets with seventh-generation adhesive (Group A: 11.54 MPa). The superior performance of eighth-generation adhesives may be attributed to their interaction with the smear layer as a bonding substrate, leaving residual smear plugs that reduce dentinal fluid flow compared with etch-and-rinse systems. In addition, certain formulations improve bond strength and reduce moisture sensitivity, important factors for stable bracket attachment in conditions complicated by saliva or blood contamination [10]. The finding that ceramic brackets demonstrated higher bond strength than metal brackets with both seventh- and eighth-generation adhesives may be explained by the presence of silane coupling agents in ceramic brackets and the possibility of mechanical or chemical bonding mechanisms enhancing adhesion to enamel [13].

Comparable trends have been reported in other studies. Reddy et al. (ceramic: 20.68 MPa; metal: 12.15 MPa) [14], Joseph and Rossouw (ceramic: 28.27 MPa; metal: 17.34 MPa) [15], Eslamian et al. (ceramic: 23.09 MPa; metal: 15.56 MPa) [16], and Odegaard and Segner (ceramic: 23 MPa; metal: 20.7 MPa) [17] reported higher SBS values than those found in the present study. Conversely, Pouyanfar et al. (ceramic: 8.98 MPa; metal: 6.07 MPa) [18] and Elsaka (ceramic: 8.93 MPa; metal: 5.63 MPa) [19] reported lower values. Such differences may be attributed to variations in bracket base design, silanization for chemical retention, bracket base technology (e.g., polycrystalline alumina with roughened bases incorporating sharp crystals or spherical glass particles), differences in adhesives and bonding agents used, application techniques, force magnitude and duration, bracket type and size, and the inclusion or omission of thermocycling in the experimental design.

In contrast to the present findings, several studies have reported higher bond strengths in metal brackets compared with ceramic brackets, including Mirzakouchaki et al. (metal: 9.99 MPa; ceramic: 7.07 MPa) [20], Arash et al. (metal: 11.15 MPa; ceramic: 7.41 MPa) [21], Pinho et al. (metal: 6.9 MPa; ceramic: 4.7 MPa) [22], and Chalipa et al. (metal: 23.01 MPa; ceramic: 10.7 MPa) [23]. These discrepancies may be attributed to differences in bracket types and base designs.

The mean difference in SBS between ceramic brackets bonded with seventh-generation adhesive (Group B) and eighth-generation adhesive (Group D) was 1.75 MPa. Similarly, the mean difference between metal brackets bonded with seventh-generation adhesive (Group A) and eighth-generation adhesive (Group C) was 1.55 MPa, indicating superior performance of the eighth-generation adhesive for both bracket types.

Comparable results have been reported by Mishra et al. (eighth generation: 28.72 N; seventh: 26.22 N) [5], Nair et al. (eighth: 34.93 N; seventh: 31.88 N) [24], Kamble et al. (eighth: 34.74 N; seventh: 31.67 N) [25], and Chauhan et al. (eighth: 40.44 N; seventh: 22.42 N) [26], all of whom found higher bond strengths with eighth-generation adhesives. This improvement is attributed to crosslinking of highly functional SiO₂ nanoparticles, which infiltrate the smear layer without completely removing it, securing smear plugs at tubule entrances, reducing dentinal fluid flow, and incorporating residual hydroxyapatite in the resin-impregnated smear and hybrid layer for additional chemical retention.

However, average in vivo bond strengths are typically about 40% lower than those measured in vitro [27]. Bond strength also decreases gradually due to aging and material storage in saliva, as reported by Boyer et al., Kao et al., and Chiba et al. [28-30]. Therefore, in vivo bond strengths are generally lower than those measured under in vitro conditions.

Limitations

The complex nature of the oral environment underscores the need for caution when extrapolating in vitro findings to clinical practice. While in vitro studies provide controlled conditions and serve as useful preliminary screening tools, they cannot fully replicate variations in temperature, humidity, pH, or the mechanical and masticatory stresses experienced in the oral cavity. In vitro experiments, however, remain valuable for guiding future in vivo research. Additionally, variations in bracket base design may contribute to differences in SBS. The storage period of extracted premolars may also have influenced enamel surface properties. Future studies with larger sample sizes are warranted to substantiate and clarify these findings.

## Conclusions

The SBS hierarchy observed in this study was highest in ceramic brackets bonded with eighth-generation adhesives, followed by ceramic brackets with seventh-generation adhesives, metal brackets with eighth-generation adhesives, and lowest in metal brackets with seventh-generation adhesives. All differences were statistically significant. Overall, eighth-generation adhesives demonstrated superior SBS compared with seventh-generation adhesives, and ceramic brackets performed better than metal brackets. The SBS values obtained were well above Reynolds’ recommended threshold, confirming that both seventh- and eighth-generation adhesives provide clinically acceptable bond strength.

Although in vitro studies offer valuable preliminary insights, caution must be exercised when extrapolating these findings to clinical practice, as the oral environment’s complex conditions, such as temperature fluctuations, humidity, pH variations, and masticatory forces, cannot be fully replicated in vitro. Additionally, storage duration may influence enamel quality. Future research with larger sample sizes and in vivo designs is necessary to validate and build upon these results.
